# Identifying Cognitive Impairment in Elderly Using Coupling Functions Between Cerebral Oxyhemoglobin and Arterial Blood Pressure

**DOI:** 10.3389/fnagi.2022.904108

**Published:** 2022-05-20

**Authors:** Wenhao Li, Guanwen Qu, Congcong Huo, Xiaoling Hu, Gongcheng Xu, Huiyuan Li, Jingsha Zhang, Zengyong Li

**Affiliations:** ^1^Key Laboratory for Biomechanics and Mechanobiology of Ministry of Education, School of Biological Science and Medical Engineering, Beihang University, Beijing, China; ^2^Beijing Advanced Innovation Center for Biomedical Engineering, Beihang University, Beijing, China; ^3^Beijing Key Laboratory of Rehabilitation Technical Aids for Old-Age Disability, National Research Center for Rehabilitation Technical Aids, Beijing, China; ^4^Key Laboratory of Neuro-Functional Information and Rehabilitation Engineering of the Ministry of Civil Affairs, Beijing, China; ^5^Department of Biomedical Engineering, The Hong Kong Polytechnic University, Kowloon, Hong Kong SAR, China

**Keywords:** cognitive dysfunction, coupling function, near-infrared spectroscopy, arterial blood pressure, tissue oxygenation index

## Abstract

**Background:**

This study aimed to assess brain oxygenation status and cerebral autoregulation function in subjects with cognitive dysfunction.

**Methods:**

The Montreal Cognitive Assessment (MoCA) was applied to divide the subjects into three groups: cognitive impairment (Group CI, 72.50 ± 10.93 y), mild cognitive impairment (Group MCI, 72.02 ± 9.90 y), and normal cognition (Group NC, 70.72 ± 7.66 y). Near-infrared spectroscopy technology and a non-invasive blood pressure device were used to simultaneously measure changes in cerebral tissue oxygenation signals in the bilateral prefrontal lobes (LPFC/RPFC) and arterial blood pressure (ABP) signals from subjects in the resting state (15 min). The coupling between ABP and cerebral oxyhemoglobin concentrations (Δ [O_2_Hb]) was calculated in very-low-frequency (VLF, 0.02–0.07 Hz) and low-frequency (LF, 0.07–0.2 Hz) bands based on the dynamical Bayesian inference approach. Pearson correlation analyses were used to study the relationships between MoCA scores, tissue oxygenation index, and strength of coupling function.

**Results:**

In the interval VLF, Group CI (*p* = 0.001) and Group MCI (*p* = 0.013) exhibited significantly higher coupling strength from ABP to Δ [O_2_Hb] in the LPFC than Group NC. In the interval LF, coupling strength from ABP to Δ [O_2_Hb] in the LPFC was significantly higher in Group CI than in Group NC (*p* = 0.001). Pearson correlation results showed that MoCA scores had a significant positive correlation with the tissue oxygenation index and a significant negative correlation with the coupling strength from ABP to Δ [O_2_Hb].

**Conclusion:**

The significantly increased coupling strength may be evidence of impaired cerebral autoregulation function in subjects with cognitive dysfunction. The Pearson correlation results suggest that indicators of brain oxygenation status and cerebral autoregulation function can reflect cognitive function. This study provides insights into the mechanisms underlying the pathophysiology of cognitive impairment and provides objective indicators for screening cognitive impairment in the elderly population.

## Introduction

As the population ages, dementia has become a global public health concern (Kisa et al., 2018). It is estimated that 115 million people will be living with dementia by 2050, which is a great challenge affecting families, communities, and health care systems around the world (Prince et al., 2015). Mild cognitive impairment is thought to lie on a functional continuum between normal cognitive aging and the earliest signs of dementia (Rombouts et al., 2005). Studies have indicated that early interventions in the early stages of dementia (i.e., mild cognitive impairment) present an opportunity to improve or maintain cognitive function to slow the trajectory of cognitive decline (Livingston et al., 2017). Without an intervention, the neurodegenerative process will cause irreversible atrophy (Raz et al., 2016). Accurate identification of cognitive dysfunction is a prerequisite to receiving these interventions. Therefore, the establishment of boundaries between normal aging and dementias using reliable, sensitive, quantitative, and objective criteria is essential for improved clinical outcomes.

Cerebral autoregulation is a protective mechanism that maintains cerebral blood flow at a relatively constant level despite fluctuations of cerebral perfusion pressure (Beek et al., 2008). Cerebral autoregulation is a frequency-dependent phenomenon that allows rapid ABP changes (<0.2 Hz) to be transmitted to cerebral blood flow, whereas slow ABP changes are filtered (Numan et al., 2014; Claassen et al., 2016). Impaired cerebral autoregulation leads to a greater dependence of cerebral blood flow on blood pressure, leaving brain tissue unprotected against the potentially harmful effects of blood pressure fluctuations (den Abeelen et al., 2014). It has been evidenced that cerebral autoregulation function is altered or impaired in patients with a variety of conditions such as diabetes (Hu et al., 2008), Parkinson's disease (Vokatch et al., 2007), and stroke (Xiong et al., 2017). Recent research suggested an interrelationship between Alzheimer's disease pathology, radiographic markers of cerebral hypoperfusion, and cerebral autoregulation (Brickman et al., 2015; Zhou et al., 2019). Therefore, it can be hypothesized that cerebral perfusion and cerebral autoregulation are altered in subjects with cognitive dysfunction.

Cerebral autoregulation assessment requires accurate and continuous measurements of cerebral blood flow (Lam et al., 2019). Near-infrared spectroscopy (NIRS) is a non-invasive neuroimaging technique that allows the continuous measurement of tissue oxygenation and hemodynamic parameters in the cerebral (Kozlová, 2018). The attributes of NIRS such as portability, tolerance of motion artifacts, and use in patients with pacemakers and metal implants have made this technique particularly suitable for the analysis of cerebral autoregulation in the elderly population (Addison, 2015). Kainerstorfer et al. (2015) demonstrated the reliability of non-invasive measurement of cerebral autoregulation in microvascular systems using NIRS. Currently, NIRS has been used to observe cerebral autoregulation in patients with subarachnoid hemorrhage (Budohoski et al., 2016), acute neurological injury (Rivera et al., 2017), and sepsis patients (Eleveld et al., 2021). Therefore, in the present study, NIRS was employed to investigate cerebral autoregulation function in subjects with cognitive dysfunction.

In past decades, various methods have been adopted for the non-invasive assessment of cerebral autoregulation in the resting state based on spontaneous fluctuations in blood pressure and cerebral blood flow. Of all the available methods to do this, transfer function analysis is the most frequent method reported in the literature to quantify cerebral autoregulation (Meel-van den Abeelen et al., 2014). Nevertheless, the cerebral autoregulation parameters calculated by transfer function analysis do not seem to differentiate between subjects with cognitive dysfunction (Gommer et al., 2012; Tarumi et al., 2014). Close attention has recently been dedicated to the study of coupling functions based on dynamical Bayesian inference, which has been used in the assessment of cerebral autoregulation function in patients with stroke and hypertension (Su et al., 2018; Li et al., 2021). A great advantage of the Bayesian method is its ability to simultaneously detect time-varying synchronization, the directionality of coupling, and time-evolving coupling functions, even in the presence of noise (Stankovski et al., 2014). This study aimed to investigate the potential of the coupling function method based on dynamic Bayesian inference for the assessment of cerebral autoregulation in subjects with cognitive dysfunction.

In this study, NIRS and non-invasive blood pressure devices were used to simultaneously measure cerebral oxygenation signals in the prefrontal cortex (PFC) and arterial blood pressure (ABP) signals from subjects in the resting state. Coupling function between the ABP and cerebral oxygenation signals was established based on dynamical Bayesian inference. Very-low-frequency (VLF, 0.02–0.07 Hz) and low-frequency (LF, 0.07–0.20 Hz) oscillation of oxyhemoglobin has shown to be robust parameter for evaluating cerebral autoregulation (Kainerstorfer et al., 2015; Eleveld et al., 2021). Spontaneous oscillations in the VLF interval are mainly associated with hemodynamic fluctuations that originate from spontaneous cortical neural activity, and the spontaneous oscillations in the LF interval are believed to reflect vasomotor and sympathetic activity (Vermeij et al., 2014). In the present study, cerebral autoregulation function in elderly subjects with cognitive dysfunction was assessed by coupling functions in the VLF and LF bands and compared with those in healthy elderly controls. Pearson correlation analysis was used to study the relationships between montreal cognitive assessment (MoCA) scores and indicators of cerebral autoregulation function. In addition, we investigated the relationship between MoCA scores and brain oxygenation status. This study provides insights into the mechanisms underlying the pathophysiology of cognitive impairment and provides objective indicators for screening cognitive impairment in the elderly population.

## Methods

### Participants

This study was performed in senior centers and the Rehabilitation Hospital, National Research Center for Rehabilitation Technical Aids. The trial was registered with the Chinese Clinical Trial Registry (registration no. ChiCTR2100053043). Written informed consent was obtained from the participants before the study. When the subject had difficulties understanding the informed consent due to cognitive dysfunction, their family provided content. All procedures performed in this study involving human participants were in accordance with the Declaration of Helsinki (as revised in 2013).

**Figure 1:**
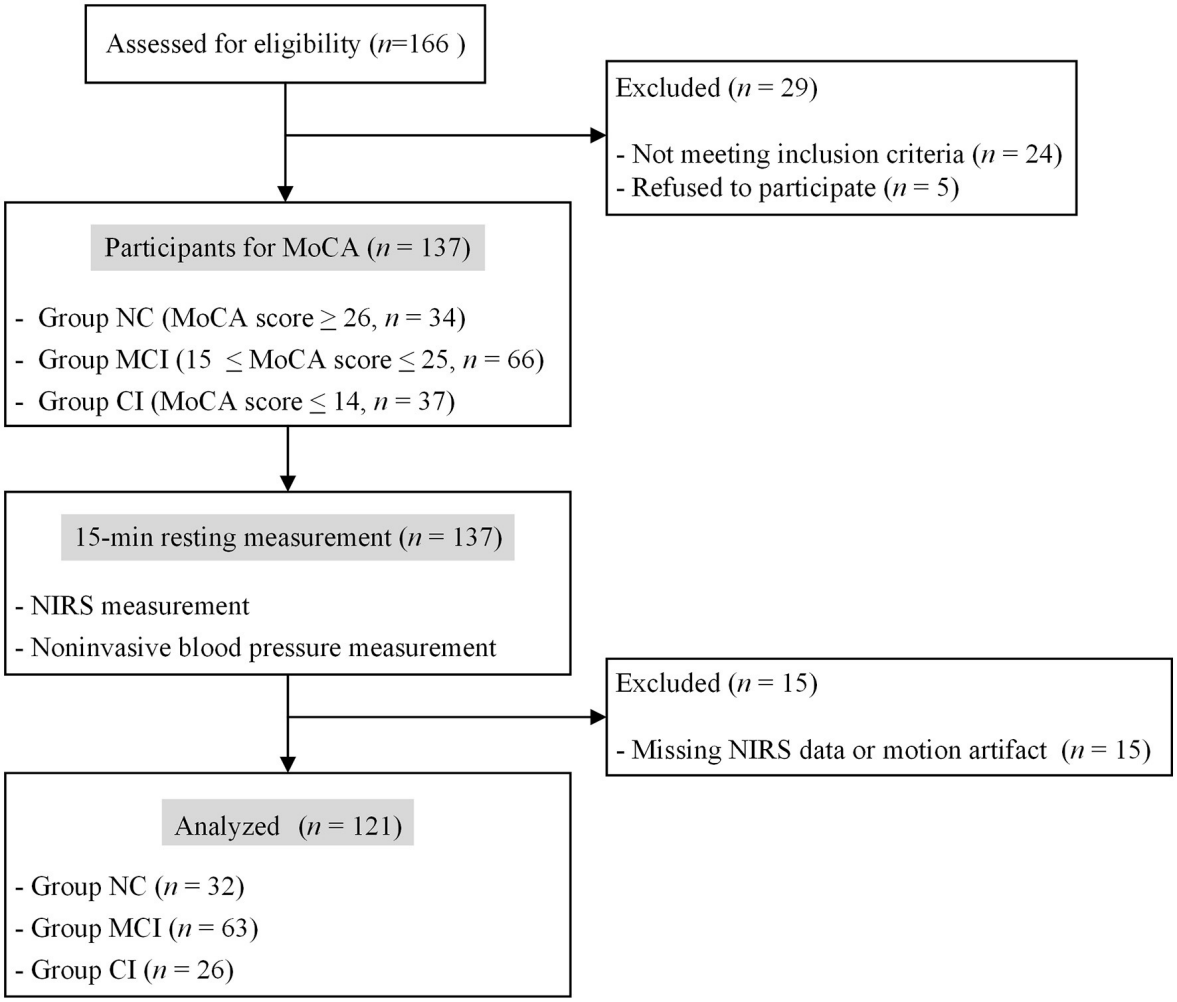
Flow chart of participant inclusion.

A total of 166 volunteers were initially enrolled in this study (Figure 1). The target group was right-handed elderly individuals aged > 50 years. The exclusion criteria during the initial enrollment were neurological illness and traumatic brain injury with any known cognitive consequences. The subjects were grouped according to the MoCA scores, which was administered by trained personnel. The subjects with MoCA scores 26 or above were categorized as normal cognition (Group NC); those with scores between 15 and 25 were categorized as mild cognitive impairment (Group MCI); and those with scores of 14 or less were categorized as cognitive impairment (Group CI).

### Data Collection

After recording basic information and MoCA scores, cerebral oxygenation and ABP signals were simultaneously collected. All measurements were non-invasive and safe for the subjects. After 5 min of rest, 15 min recordings were made for (1) ABP that were continuously measured with finger-pulse photoplethysmography at a sampling rate of 1,000 Hz (CNAP™ Monitor 500, CNSystems Medizintechnik AG, Graz, Austria); (2) cerebral oxygenation data that were measured via NIRS (ECO-N17-C25L, Enginmed Bio-Medical Electronics, Suzhou, China) at a sampling rate of 20 Hz. Each sensor of the ECO-N17-C25L consisted of a light-emitting diode and two PIN diodes. The light-emitting diode component worked with three-wavelengths (760, 810, and 840 nm) and served as the source of emitted light, whereas the PIN diodes served as the detectors. The distances between the light source and the two detectors were 30 and 40 mm. The differential spacing of the receiving detectors provided spatial resolution to distinguish signals from cerebral and extracerebral tissue. The probes were positioned over the PFC area (LPFC/RPFC) and then wrapped around the forehead with an elastic bandage to block ambient light.

### Signals and Preprocessing

The ECO-N17-C25L used a spatially resolved spectroscopy algorithm to calculate the concentration changes in oxygenated and deoxygenated hemoglobin concentrations (Δ [O_2_Hb] and Δ [HHb], respectively) compared with their original values in human tissue. It has been shown that this algorithm is little influenced by either background absorption or overlying tissues (Teng et al., 2006; Han and Zhang, 2016). The tissue oxygenation index (TOI) is an indicator that characterizes the brain oxygenation status, which directly reflect the dynamic balance between oxygen supply and consumption in regional tissue (Jin et al., 2021). The TOI value can be derived from the ratio of tissue oxygenated hemoglobin concentrations to total hemoglobin concentration in blood flow within venous, arterial, and cerebral cortical tissue, where the total hemoglobin concentration is the sum of the [O_2_Hb] and [HHb] concentrations (Naulaers et al., 2007). Mathematically, TOI (%) can be expressed as follows:


(1) $$\begin{array}{r} \text{TOI }=\;\frac{{\text{O}}_{2}\text{Hb}}{{\text{O}}_{2}\text{Hb }+\text{HHb}}\;\times \;100\% \end{array}$$

Moving average and cubic spline interpolation methods were used to eliminate noise-like abrupt spikes and motion artifacts in the NIRS signal, respectively (Scholkmann et al., 2010). The window width of the moving average method was 5 s. To achieve a uniform time basis, the raw ABP signal was downsampled to 20 Hz.

### Data Analysis

**Figure 2:**
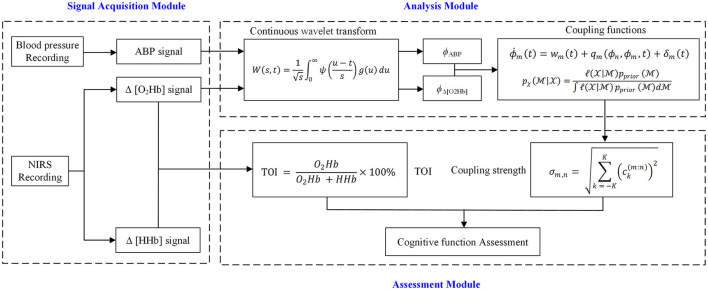
Overview of modeling of interaction between NIRS and ABP signals.

In the present study, the cerebral autoregulation function was assessed by investigating interactions between cardiac oscillations and slow oscillations in the cerebral. An overview of the modeling of the interaction between brain activity and ABP is shown in Figure 2. In the first step, the phase time series of NIRS and ABP signals were extracted by continuous wavelet transform. In the second step, the interactions between extracted components were studied by a coupled-phase-oscillator model based on dynamical Bayesian inference. Finally, the coupling direction and coupling strength were calculated to quantify the coupled systems. All these methods were explained below.

#### Dynamic Phase Extraction for Coupling Function Analysis

In the present study, the continuous wavelet transform was used to extract spontaneous oscillations of NIRS and ABP signals in various characteristic frequency bands. The continuous wavelet transform is a time–frequency analysis method, which uses the logarithmic scale for the frequency, thus low frequencies have higher resolutions. The continuous wavelet transform is given by the equation:


(2) $$\begin{array}{c} W(s,t)\;=\;\frac{1}{\sqrt{s}}\int_{0}^{\infty }\psi (\frac{u\;-\;t}{s})g(u)du \end{array}$$

where *W*(*s, t*) is the wavelet coefficient, *g*(*u*) is the time series, and ψ is the mother wavelet, scaled by the factor *s* and translated in time by *t*. The complex Morlet wavelet $\psi \left(u\right)\;=\;\frac{1}{\sqrt[{4}]{\pi }}e^{-i2\pi \mu }e^{-\mu^{2}/2}$ (with *i* the imaginary unit) was chosen to be the mother wavelet because it maximizes joint time-localization and frequency resolution (Stefanovska et al., 1999).

The phases of Δ [O_2_Hb] signal (ϕ_Δ[*O*2*Hb*]_) was extracted in the VLF (0.02–0.07 Hz) and LF (0.07–0.2 Hz) range. The phase extraction of the heart activity from the ABP signal (ϕ_*ABP*_) was 0.6–2 Hz (Stefanovska, 2007). The signals extracted from these intervals are periodic, enabling the underlying oscillatory processes and their interactions to be studied effectively through phase dynamics, and leading to extraction of phase-to-phase cross-frequency couplings (Stankovski et al., 2017a).

#### Coupling Functions Using Dynamical Bayesian Inference

The interactions were modeled with cross-frequency coupling based on dynamical Bayesian inference. Coupling functions prescribe the physical rule specifying how the inter-oscillator interactions occur. To learn about influence of each oscillator on the others, the system was decomposed into a group of phase oscillators which interact. Their decomposition can describe the functional contribution from each separate subsystem within a single coupling relationship (Stankovski et al., 2016). This system can be defined by two differential stochastic equations (Stankovski et al., 2017b):


(3) $$\begin{array}{c} {\overset{\cdot }{\phi }}_{m}(t)\;=\;w_{m}(t)+q_{m}(\phi_{n},\phi_{m},t)+\delta_{m}(t) \end{array}$$

with *m* = 1, *n* = 2. where *w*_*m*_ and ϕ_*m*_ are the natural frequency and phase of oscillator *m*, δ_*m*_(*t*) is Gaussian white noise, and *q*_*m*_(ϕ_*n*_, ϕ_*n*_, *t*) is the coupling function describing the influence of oscillator *n* on the phase of oscillator *m*.

The theorem of dynamical Bayesian inference is summarized in Stankovski et al. (2012):


(4) $$p_{\chi }\left(ℳ|X\right)\;=\;\frac{\ell \left(X|ℳ\right)p_{prior}\left(ℳ\right)}{\int \ell \left(X|ℳ\right)p_{prior}\left(ℳ\right)dℳ}$$

where $p_{\chi }\left(ℳ|X\right)\;$ is the conditional probability of observing the data X given the hypothesized parameters M. $p_{prior}\left(ℳ\right)$ is the probability of M before observing the data X. $p_{\chi }\left(ℳ|X\right)\;$ is known as the posterior probability–the probability that the hypothesized parameters are correct given X and the prior probability $p_{prior}\left(ℳ\right)$.

#### Quantitative Measures

To simplify quantitative comparisons obtained results, the coupling strength and coupling direction are calculated. The coupling strength gives a quantitative measure of the information flow between the coupled systems and is an important indicator to characterize the magnitude and the extent of the coupling relationship (Stankovski et al., 2017b). A higher coupling strength value indicates that the fluctuations in one oscillation are more direct in transferring amplitude changes to the other. The coupling direction represents the predominant direction of the coupling function. The strength *CS*_*m, n*_ of the coupling from the oscillator *m* to *n* is defined as:


(5) $$\begin{array}{r} CS_{m,n}=\;\sqrt{\sum_{k\;=\;-\;K}^{K}{\left(c_{k}^{\left(m:n\right)}\right)}^{2}} \end{array}$$

The directionality index *CD* represents the predominant direction of the coupling function, which is defined as (Stankovski et al., 2012):


(6) $$\begin{array}{r} CD\left(t\right)\;=\;\frac{CS_{n,m}\;-\;CS_{m,n}}{CS_{n,m}\;+\;CS_{m,n}} \end{array}$$

If *CD* ∈[−1, 0] (*CD* ∈[0, 1]), the *n* (*m*) drives the *m* (*n*). The result of the *CD* value calculated from Equation (6) is >0. Therefore, only the coupling functions in the direction from ABP to Δ [O_2_Hb] in the VLF and LF interval were discussed in the present study.

### Statistical Analysis

Age, body mass index, sex, blood pressure, and MoCA scores are expressed as the means and standard deviation. The Kolmogorov–Smirnov and Levene tests were applied to test variance normality and homogeneity of the data at the group level. Significant intergroup differences in TOI and coupling strength were assessed by one-way ANOVA. Bonferroni's *t*-test was used for the intergroup pairwise comparisons. Three comparisons between the groups were designed (Group NC vs. Group MCI, Group NC vs. Group CI, and Group MCI vs. Group CI). Therefore, the corrected statistical significance was defined as *p* < 0.0167 (*p* < *p*_original_/3). The associations between MoCA scores, TOI, and coupling strength were assessed by Pearson's correlation analysis. A difference of *p* < 0.05 was considered statistically significant. Receiver–operator characteristic analysis with Youden's J statistic was used to test the sensitivity and specificity and determine the optimal threshold value for the TOI and cerebral autoregulation indices to differentiate subjects with mild cognitive impairment from those with normal cognition.

## Results

### Demographic and Cognitive Test Results

The demographic characteristics and cognitive test results for each group are shown in Table 1. The demographics of Groups NC, MCI, and CI, including age, sex, body mass index, and blood pressure were not significantly different among the three groups. The groups did have significantly different MoCA scores.

**Table 1:** Basic information of the participants.

**Characteristic**	**Group NC**	**Group MCI**	**Group CI**	***p*****-value** **(NC vs. MCI)**	***p*** **-value (NC vs. CI)**	***p*****-value** **(MCI vs. CI)**
Age (year)	70.72 ± 7.66	72.02 ± 9.90	72.50 ± 10.93	0.535	0.484	0.829
Gender (male/female)	9/23	31/32	13/13	0.051	0.096	0.945
Body mass index	22.00 ± 1.92	22.89 ± 3.00	22.52 ± 2.33	0.123	0.456	0.550
MoCA score	26.94 ± 1.54	20.60 ± 3.42	10.62 ± 3.16	<0.001*	<0.001*	<0.001*
Systolic blood pressure (mm Hg)	120.55 ±17.18	124.60 ± 19.92	129.14 ± 24.60	0.361	0.113	0.341
Diastolic blood pressure (mm Hg)	70.41 ± 8.60	69.93 ± 12.70	68.20 ± 15.79	0.860	0.506	0.555

Values are presented as means and standard deviations. *Indicates p < 0.05.

### Group-Dependent Variation in TOI

**Figure 3:**
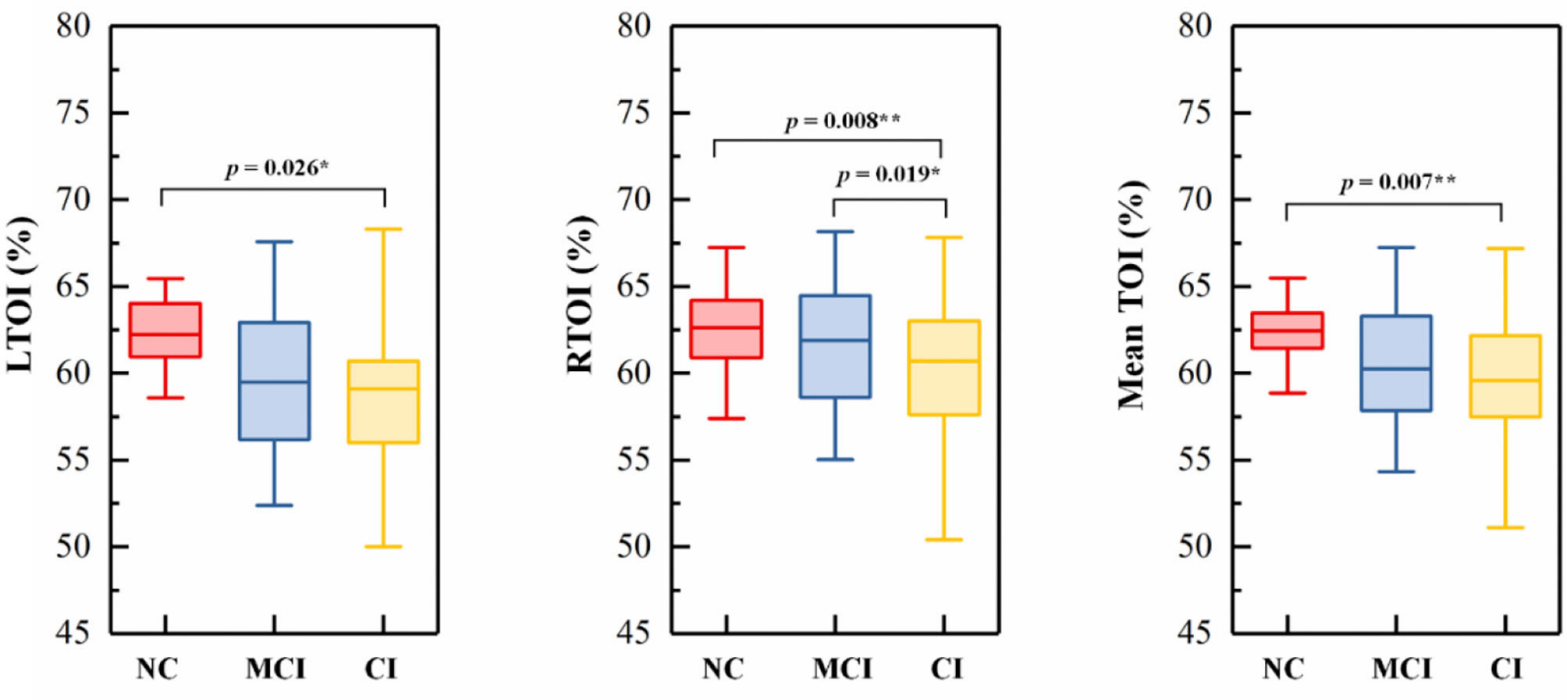
Box-plots picturing the distributions of **(A)** LTOI, **(B)** RTOI, and **(C)** mean TOI. In each boxplot, the NC group (NC) is indicated by red rectangles, the MCI group (MCI) by blue rectangles, and the CI group (CI) by yellow rectangles. The line connectors on the tops of individual panels indicate cases where the difference between two boxplot distributions was statistically significant. *p* < 0.05 are marked with *, *p* < 0.0167 are marked with **.

The TOI in the left and right PFC (LTOI/RTOI) of each subject was calculated by averaging the TOI values in the time domain over the acquisition period (15 min). The averaged TOI of the bilateral PFC was expressed as Mean TOI. An example of the typical curves of original NIRS data and TOI was shown in Supplementary Material. Figure 3 shows the comparison of the TOI values among the three groups. The result shows that the RTOI and mean TOI values were significantly lower in Group CI than in Group NC.

### Coupling Strength

**Figure 4:**
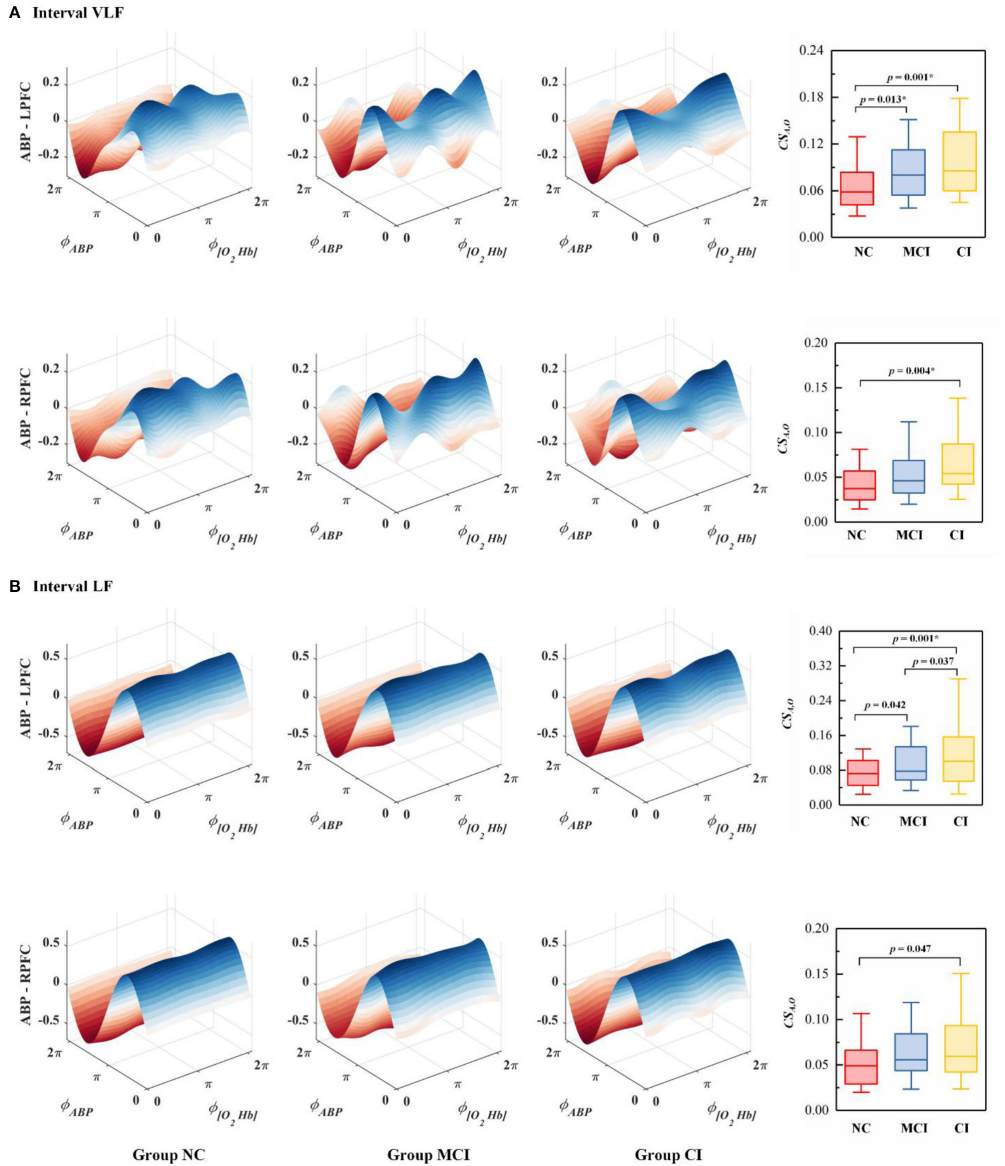
The average coupling functions from all subjects within the group between ABP and Δ [O_2_Hb] in the **(A)** VLF interval and **(B)** LF interval. The coupling between ABP and Δ [O_2_Hb] in the bilateral PFC is denoted as ABP - LPFC and ABP - RPFC. ϕ_*ABP*_ and ϕ_[*O*2*Hb*]_ represent the dynamical phase information of ABP and Δ [O_2_Hb] signal, respectively. Each boxplot shows the coupling strength distribution of a specific coupling relationship in the resting state indicated by the NC (Group NC), MCI (Group MCI), or CI (Group CI). The NC group is indicated by red rectangles, the MCI group by blue rectangles, and the CI group by yellow rectangles. The line connectors on the tops of individual panels indicate cases where the difference between two boxplot distributions was statistically significant. *p* < 0.0167 are marked with *.

The phase-to-phase coupling functions between ABP and Δ [O_2_Hb] were reconstructed, quantified, and compared. Figure 4 presents the specific-frequency coupling function between ABP and Δ [O_2_Hb] in each group and the corresponding coupling strength. In the VLF interval, Group CI exhibited significantly higher coupling from ABP to Δ [O_2_Hb] (*CS*_*A,O*_) in the LPFC and RPFC than Group NC. In the VLF interval, the *CS*_*A,O*_ was significantly higher in Group MCI than in Group NC in LPFC. In the LF interval, the *CS*_*A,O*_ in the LPFC was significantly higher in Group CI than in Group NC.

### Correlation Analysis

**Figure 5:**
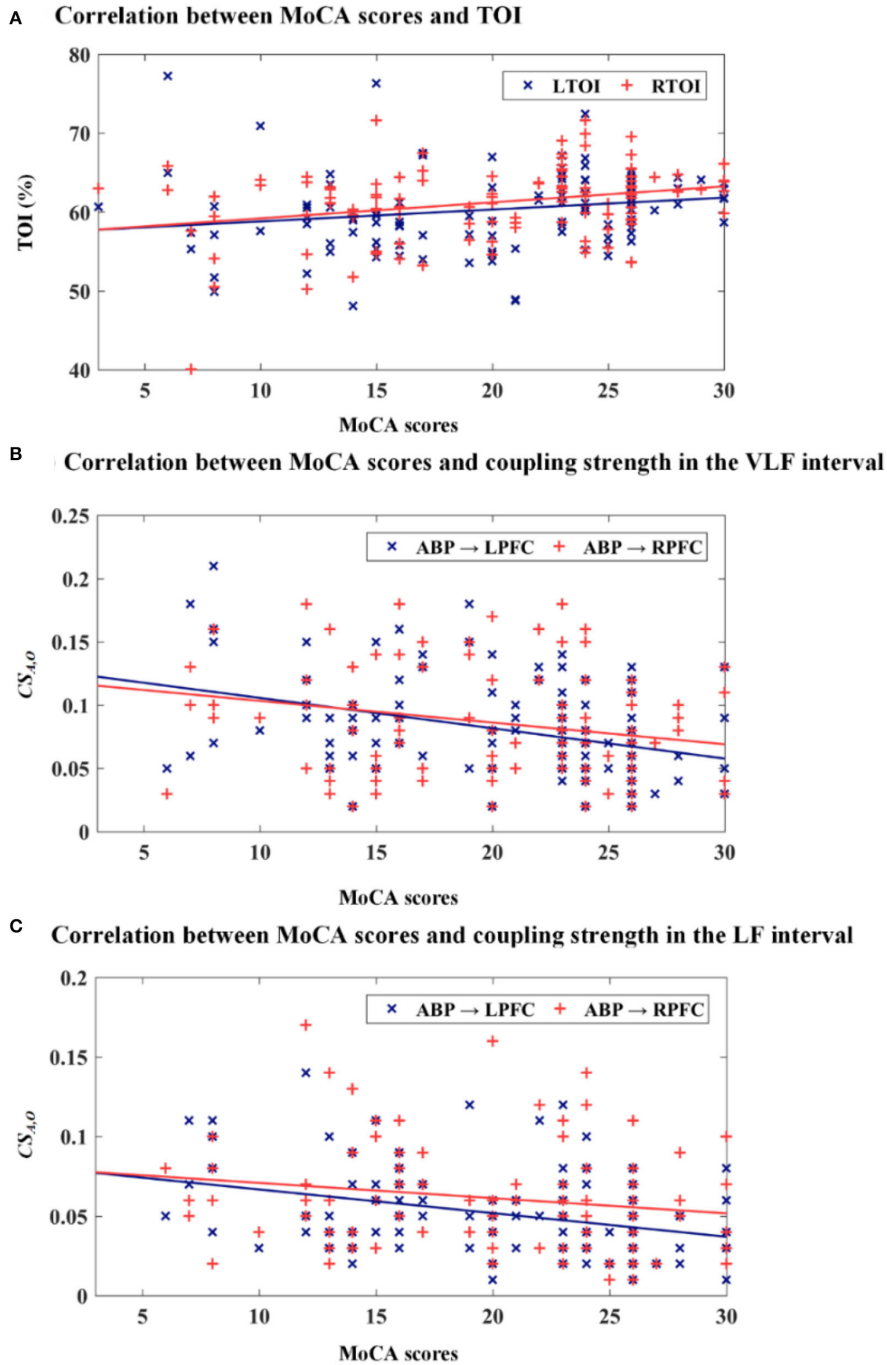
Scatterplots of TOI vs. MoCA scores and coupling strength vs. MoCA scores. **(A)** MoCA scores vs. TOI, **(B)** MoCA scores vs. *CS*_*A,O*_ in the VLF interval, and **(C)** MoCA scores vs. *CS*_*A,O*_ in the LF interval.

Scatterplots of TOI vs. MoCA scores and coupling strength vs. MoCA scores are presented in Figure 5. The correction between the MoCA scores, TOI, and *CS*_*A,O*_ in the bilateral PFC is presented in Table 2. MoCA scores show a statistically significant positive correlation with LTOI and RTOI. MoCA scores are significantly negatively correlated with *CS*_*A,O*_ in the LPFC and RPFC interval VLF and LF.

**Table 2:** Correlations between the MoCA scores, TOI, and *CS*_*A,O*_ at rest in the bilateral PFC.

**Variables**		**MoCA**	
		***r***	***p***
TOI	LTOI	0.199	0.029*
	RTOI	0.282	0.002*
*CS*_*A,O*_	ABP → LPFC (VLF)	−0.390	<0.001*
	ABP → LPFC (LF)	−0.378	<0.001*
	ABP → RPFC (VLF)	−0.328	<0.001*
	ABP → RPFC (LF)	−0.226	0.014*

p < 0.05 are marked with ^*^.

### Receiver–Operator Characteristic Analysis

A receiver–operator characteristic analysis with the corresponding area under the curve was performed on TOI and coupling strength values to determine the optimal threshold value for distinguishing subjects with mild cognitive impairment from those with normal cognition. The discriminant validity for the detection of mild cognitive disorder of the mean TOI and coupling strength between the bilateral PFC [receiver–operator characteristic area under the curve (95% confidence interval)]: TOI [0.65 (0.54, 0.74)] and coupling strength [0.64 (0.53, 0.74)]. The optimal threshold value of the mean TOI in the bilateral PFC was 60% (sensitivity = 54.0%, specificity = 84.4%), and the optimal threshold value of the averaged *CS*_*A,O*_ in the bilateral PFC was 0.05 (sensitivity = 77.4%, specificity = 41.9%). TOI values below the optimal threshold value and coupling strength values above the optimal threshold value appeared to be closely associated with the diagnosis of mild cognitive impairment.

## Discussion

In this research, cerebral autoregulation function was assessed by a coupling function based on dynamical Bayesian inference. The main findings of this study were as follows: (1) TOI was significantly reduced on both sides of the PFC in subjects with cognitive dysfunction; (2) cerebral autoregulation function was impaired in subjects with cognitive dysfunction. The main strengths of this study include the application of a coupling function, which provides insights into the mechanisms underlying the pathophysiology of cognitive impairment. The present study describes the potential mechanism and clinical implications of our findings.

Good oxygenation status is a guarantee for nerve cells to maintain structural integrity and normal function of the brain. A large body of evidence indicates that cerebral hypoperfusion is one of the earliest pathological signs in the development of cognitive failure (de la Torre, 2004). Meta-analyses demonstrated clear abnormalities in cerebral hemodynamic and oxygenation parameters in patients with mild cognitive dysfunction, even at an early stage of cognitive decline (Beishon et al., 2017). It is clinically important to monitor the oxygenation status of cerebral tissue in real-time, detect abnormalities, and initiate timely intervention measures. TOI is an indicator of the oxygen saturation in regional tissues, and variations in TOI can reflect changes in cerebral blood flow to some extent (Jin et al., 2021). Tarumi et al. (2014) found that TOI was reduced at rest in subjects with mild cognitive dysfunction compared with healthy controls. Consistent with the literature, reduced TOI on both sides of the PFC was observed in participants in Group MCI and Group CI compared to controls. This result suggested that cerebral perfusion was reduced in subjects with cognitive dysfunction compared to healthy elderly adults. A possible explanation for this result may be the reduction in brain metabolic demand that parallels cognitive decline. Another possible explanation for this is that chronic brain hypoperfusion in elderly individuals leads to neuronal damage and eventually to neurodegenerative tissue atrophy (de la Torre, 2004, 2008).

A previous study has suggested that sustained mild hypoxia reduces steady-state cerebral blood flow, and continuously impairs cerebral autoregulation (Nishimura et al., 2010). Cerebral autoregulation allows the maintenance of relatively stable cerebral perfusion and brain tissue oxygenation against changes in blood pressure through complex myogenic, neurogenic, and metabolic mechanisms (Addison, 2015). The VLF and LF bands are in the frequency range where cerebral autoregulation is considered operative. The current interpretation of the coupling function metric assumes that pressure fluctuations are more liable to induce linear and pressure-synchronized cerebral blood flow fluctuations with greater magnitude in the condition of disturbed cerebral autoregulation. Therefore, higher values of *CS*_*A,O*_ are considered to reflect greater oscillations of Δ [O_2_Hb] in response to changes in ABP, that is, poorer damping of the effectiveness of cerebral autoregulation, which represents poorer cerebral autoregulation function, and vice versa. A significantly higher *CS*_*A,O*_ was observed in Groups CI and MCI than in healthy controls in the VLF and LF intervals. These results indicated that cerebral autoregulation function was impaired in subjects with mild-to-severe cognitive dysfunction.

In the present study, the significantly increased *CS*_*A,O*_ in the VLF interval suggested that one of the mechanisms for impaired cerebral autoregulation in cognitive dysfunction patients might involve alterations in autonomic nervous activities. The continuous activity of the autonomous nervous system serves to maintain the basal level of vessel contraction. The nerves release substances that affect the activities of smooth muscles, leading to changes in the vessel radii and resistance (Shiogai et al., 2010). To maintain flow in the autoregulated range of blood pressure, cerebral resistance vessels undergo vasoconstriction during hypertension and vasodilatation during hypotension. Therefore, failure of vasoconstriction and/or vasodilatation may result in cerebral autoregulation disruption rendering the brain more susceptible to fluctuations in blood pressure (Gommer et al., 2012). In the LF interval, *CS*_*A,O*_ was significantly increased in Group CI. This appears to suggest that there is impaired myogenic activity regulation in the PFC in subjects with cognitive dysfunction. This may be related to parasympathetic depression and sympathetic exacerbation in participants with cognitive dysfunction (Toledo and Junqueira, 2008). The significant difference in coupling function was mainly distributed in the LPFC, which may be related to age-related neurodegeneration preferentially affecting the left hemisphere (Thompson et al., 2003).

In the present study, the significant correlation between TOI and MoCA scores indicates the sensitivity of cognitive function to brain oxygenation in elderly individuals. Pearson correlation analysis showed significant negative correlations between MoCA scores and *CS*_*A,O*_ in the VLF and LF intervals. This result suggests that *CS*_*A,O*_ could characterize cerebral autoregulation function changes. The results of receiver–operator characteristic analysis support the finding that value of TOI and *CS*_*A,O*_ can be used as objective indicators for screening cognitive impairment in the elderly population.

## Limitations

This study assesses brain oxygenation status and cerebral autoregulation function in subjects with cognitive dysfunction. However, the different types of cognitive dysfunction were not further classified in our study due to the relatively small sample size. In future research, more indicators and a larger sample size could be adopted to investigate the relationships between NIRS-related parameters and different types of cognitive impairment.

## Conclusion

In this pilot study, the effects of cognitive dysfunction on cerebral autoregulation function were investigated by a coupling function based on dynamic Bayesian inference. In the VLF and LF intervals, increased *CS*_*A,O*_ in Group CI and MCI indicated that cerebral autoregulation function was impaired in subjects with cognitive dysfunction. The Pearson correlation results suggested that indicators of cerebral oxygenation status and cerebral autoregulation function can reflect cognitive function. This study provides insights into the mechanisms underlying the pathophysiology of cognitive impairment. Although the method is not yet ready for large-scale application, this study provides an objective indicator for the screening of cognitive impairment in the elderly population, and with the development of NIRS and ABP techniques, the method is expected to enable large-scale community screening and routine clinical monitoring in the future.

## Data Availability Statement

The original contributions presented in the study are included in the article/Supplementary Material, further inquiries can be directed to the corresponding author/s.

## Ethics Statement

The studies involving human participants were reviewed and approved by Rehabilitation Hospital, National Research Center for Rehabilitation Technical Aids. The patients/participants provided their written informed consent to participate in this study.

## Author Contributions

WL: conceptualization, methodology, and writing—original draft. GQ and HL: data curation. CH: data curation and writing—reviewing and editing. GX: writing—reviewing and editing. XH: investigation and project administration. JZ: project administration. ZL: supervision, funding acquisition, and writing—reviewing and editing. All authors contributed to the article and approved the submitted version.

## Funding

This project was supported by the National Key Research and Development Project (2020YFC2004200), National Natural Science Foundation of China (Grant No. 11732015 and 61675013), and the Key Research and Development Project of Jiangxi Province (20202BBGL73057).

## Conflict of Interest

The authors declare that the research was conducted in the absence of any commercial or financial relationships that could be construed as a potential conflict of interest.

## Publisher's Note

All claims expressed in this article are solely those of the authors and do not necessarily represent those of their affiliated organizations, or those of the publisher, the editors and the reviewers. Any product that may be evaluated in this article, or claim that may be made by its manufacturer, is not guaranteed or endorsed by the publisher.
