# Supplementary material for: Identifying Cognitive Impairment in Elderly Using Coupling Functions Between Cerebral Oxyhemoglobin and Arterial Blood Pressure
Source: Front Aging Neurosci. 2022 May 20;14:904108. doi: 10.3389/fnagi.2022.904108 (PMC9163710; doi:10.3389/fnagi.2022.904108)
Supplement: Supplementary file 1 [file Table_1.DOCX]

**Figure S1** presented an example of the typical curves of original NIRS data and TOI in LPFC.


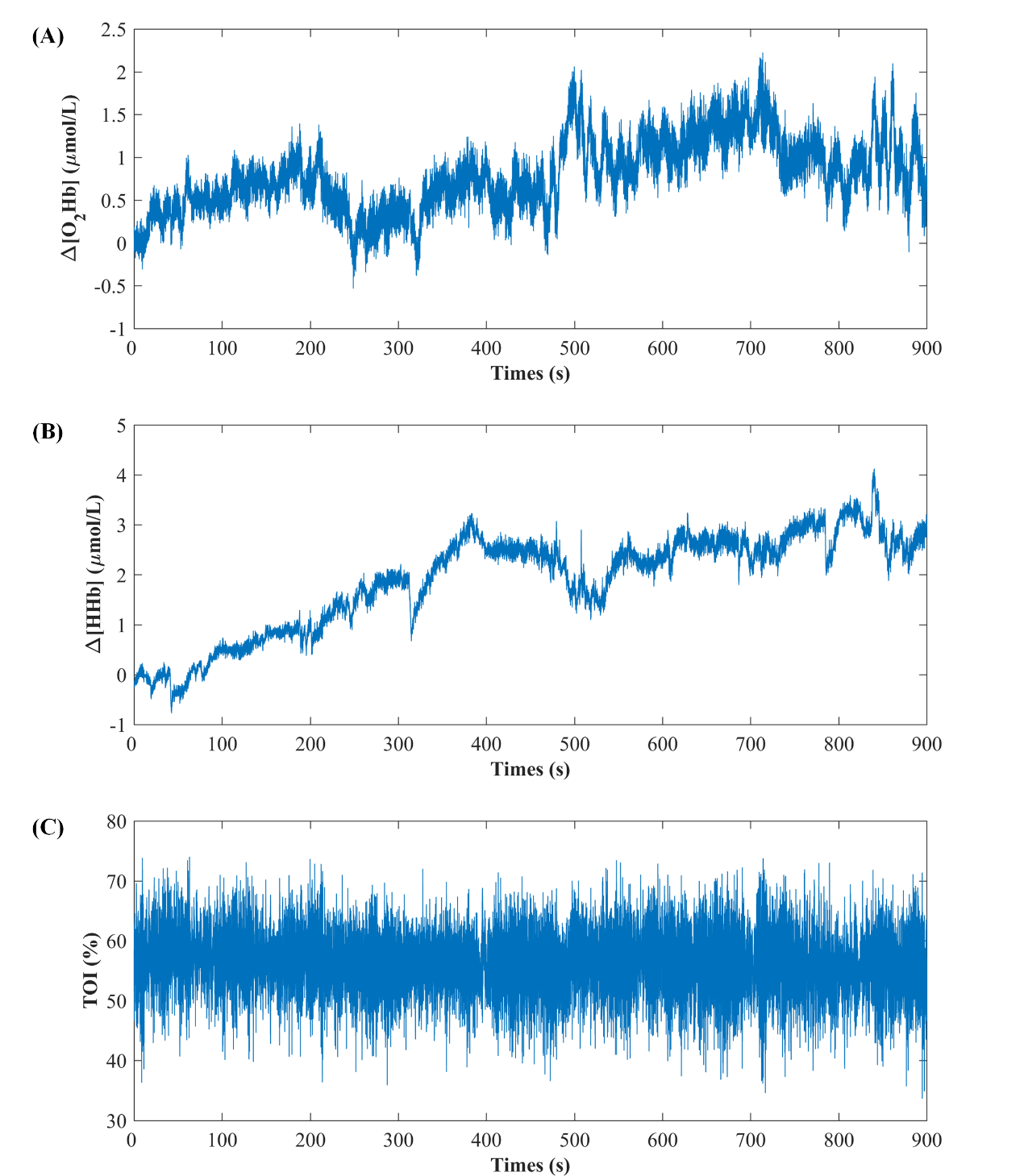


**Figure S1**. An example of the typical curves in LPFC. (A) original Δ [O_2_Hb] signal, (B) original Δ [HHb] signal, and (C) TOI.
